# Human Mammospheres Secrete Hormone-Regulated Active Extracellular Vesicles

**DOI:** 10.1371/journal.pone.0083955

**Published:** 2014-01-03

**Authors:** Esperanza Gonzalez, Marco Piva, Eva Rodriguez-Suarez, David Gil, Felix Royo, Felix Elortza, Juan M. Falcon-Perez, Maria dM. Vivanco

**Affiliations:** 1 Metabolomics CIBERehd, Derio, Spain; 2 Cell Biology and Stem Cells Unit, CIBERehd, Derio, Spain; 3 Proteomics Platform, ProteoRed-ISCIII, CIBERehd, Derio, Spain; 4 Structural Biology, CIC bioGUNE, Derio, Spain; 5 IKERBASQUE, Basque Foundation for Science, Bilbao, Spain; University of Nebraska Medical Center, United States of America

## Abstract

Breast cancer is a leading cause of cancer-associated death worldwide. One of the most important prognostic factors for survival is the early detection of the disease. Recent studies indicate that extracellular vesicles may provide diagnostic information for cancer management. We demonstrate the secretion of extracellular vesicles by primary breast epithelial cells enriched for stem/progenitor cells cultured as mammospheres, in non-adherent conditions. Using a proteomic approach we identified proteins contained in these vesicles whose expression is affected by hormonal changes in the cellular environment. In addition, we showed that these vesicles are capable of promoting changes in expression levels of genes involved in epithelial-mesenchymal transition and stem cell markers. Our findings suggest that secreted extracellular vesicles could represent potential diagnostic and/or prognostic markers for breast cancer and support a role for extracellular vesicles in cancer progression.

## Introduction

Estrogen is essential to normal mammary gland development, where it is implicated in epithelial cell proliferation and differentiation [1]. In breast cancer, approximately three out of four cases express the estrogen receptor (ER) and, consequently, tamoxifen, an ER antagonist, has been used for many years as hormonal therapy [2]. One of the most important prognostic factors for survival is the early detection of the disease, which is most often achieved through mammographic screening followed by core tissue biopsies. Therefore, less invasive methods would be highly advantageous for the diagnosis and prognosis of breast cancer and the subsequent management of individual patients. The cancer stem cell hypothesis postulates that subpopulations of cancer stem (or tumor-initiating) cells drive and maintain many types of tumor [3]. It has been shown that culture of cells as non-adherent spheres allows for propagation of stem/progenitor cells from different tissues, including the mammary gland [4]. Normal and tumor stem cells may share certain signaling pathways and, therefore, the study of normal stem cell features may lead to an understanding of the signals that are subverted during tumorigenesis [5].

Recently, small membranous vesicles of different cellular origins, referred to as extracellular vesicles (EVs), have been found in different body fluids, including blood (reviewed in [6]). The biological relevance of EVs has been demonstrated in many different processes, including intercellular communication, coagulation, immunological responses and tumor progression [7], raising expectations that EVs may provide a new source for the identification of biomarkers [8]. EVs have been found to be released by several cell types, including breast cancer cells [9], [10], and they have been implicated in the dissemination of multidrug resistance phenotype [11], [12], enhanced cellular proliferation and invasion capacity [13] and induced transformation of normal cells [14]. These vesicles have also been shown to promote the adhesion of breast epithelial cells in culture [15], [16] and recently, they were implicated in the stimulation of breast cancer cell migration through a complex inter-cellular communication process that implies the secretion of EVs by one cell type from the tumor stroma, the capture and modification/load and further secretion of activated EVs by recipient breast cancer cells [17]. All these reports suggest that EVs play an important role in the establishment and development of breast cancer. In addition, the EV features -involvement in intercellular signaling at different levels and their presence in body fluids- imply that they could be potentially useful as a source of minimally invasive markers of disease and/or convenient tools to monitor the response to treatment in different pathologies. Since cells with characteristics of stem cells can be targets of transformation, we examined the secretion of EVs by mammospheres; i.e. cell populations enriched for breast stem/progenitor cells. In this report we provide, for the first time, ultra structural, biochemical and proteomic evidence that demonstrates the secretion of EVs by primary human breast epithelial cell cultures, and that this secretion is sensitive to hormone treatment. Furthermore, we show that these EVs are captured by different cell types, and are able to increase cell proliferation and alter the expression of genes involved in stem cell maintenance and epithelial-mesenchymal transition.

## Materials and Methods

### Ethics statement

All patients were fully informed and provided written informed consent and the “Ethics Committee of Clinical Investigation of Euskadi” approved the procedures.

### Reagents

All media and reagents for tissue culture were purchased from Invitrogen (Carlsbad, CA). All other reagents were from Sigma-Aldrich, unless stated otherwise (St. Louis, MO). Monoclonal antibodies were: anti-CD81 (JS81), anti-early endosome antigen 1 (EEA1), anti-Flotillin-1 (clone 18), anti-RAB11 (clone 47), anti-ubiquitin (clone 6C1.17) and anti-BiP/GRP78 from BD Biosciences (Mountain View, CA), anti-CD13 (3D8) from Santa Cruz Biotech., Inc (Santa Cruz, CA), anti-human CD63 (H5C6) from Developmental Studies Hybridoma Bank (Iowa City, IA) and anti-CD133/Prominin1 (clone W6B3C1) from Miltenyi Biotec (Auburn, CA). Rabbit polyclonal antibodies were: anti-catalase and anti-annexin A2 from Abcam (Cambridge, UK) and anti-MFG-E8 (M-135) from Santa Cruz Biotech., Inc (Santa Cruz, CA). Horseradish peroxidase (HRP)-conjugated secondary antibody was from GE-Healthcare (Buckinghamshire, UK).

### Human tissue collection and primary mammosphere culture

Normal breast tissue was obtained from women undergoing reduction mammoplasties with no previous history of breast cancer. The breast tissue was immediately processed as previously described to yield a predominantly single cell suspension and primary mammosphere culture were performed [18]. Breast epithelial cells were grown in suspension in phenol red free DMEM:F-12 medium with GlutaMAX, supplemented with B27, 20 ng/ml EGF, 20 ng/ml bFGF and 1% penicillin/streptomycin at 37°C in 5% CO_2_. Single cell suspensions were plated at a density of 50.000 cells/ml in 75 cm^2^ ultralow attachment flasks (Corning) and treated with ethanol, as control, 10^−8^ M 17-β-estradiol (estrogen) or 10^−7^ M 4-OH-tamoxifen (tamoxifen). After 7 days, mammospheres were collected by centrifugation at 400 *g* and EVs were isolated.

### Production and isolation of extracellular vesicles

For EVs production, supernatant from mammosphere cultures of primary breast epithelial cells isolated from human biopsies (as described in [19]) or MDA-MB-468 cells was collected and vesicles secreted into the medium for six days were purified by filtration and ultracentrifugation procedure as previously described [20]. For EV production, 60 ml of supernatant from primary human mammosphere cultures were collected by centrifugation and material secreted into the medium was purified; briefly, culture supernatant was collected and centrifuged at 500 *g* for 10 min to remove cells. The resultant supernatant was subjected to filtration through 0.22 µm pore filters, followed by ultracentrifugation at 10.000 *g* and 100.000 *g* for 30 min and 60 min, respectively. The resulting pellets were washed with PBS and again ultracentrifuged at 100.000 *g* for 60 min. The final pellet of EVs was resuspended in 1/1000^th^ of the original volume of the culture supernatant, and stored at −80°C. Total protein content of EV preparations was 13.50±2.93 µg (n = 6), leading to an estimate of 4.51 pg/cell of EV production.

### Tryptic digestion

10 µg of protein from EVs isolated from ethanol-, estrogen- or tamoxifen-treated primary mammospheres was lyophilized and prepared in 50 mM ammonium bicarbonate (pH 8.5) with 0.05% Rapigest™ to assist in re-dissolving the lyophilized peptides. The samples were incubated at 60°C for 15 min and reduced in the presence of 10 mM dithiothreitol at 60°C for 30 min. The protein was alkylated in the dark in the presence of 50 mM iodoacetamide at room temperature for 30 min. Proteolytic digestion was initiated by trypsin at a concentration 1∶10 and incubated overnight at 37°C. To hydrolyze the Rapigest surfactant 2 µl of HCl was added to the sample and incubated at 37°C for 30 min, centrifuged 30 min at 10.000 *g* and the supernatant was recovered.

### Liquid Chromatography-Mass Spectrometry (LC-MS^E^)

Proteins were identified by direct analysis of the reaction mixture described above. All analyses were performed in triplicate (500 ng of material in each injection) using a NanoAcquity UPLC and Q-ToF Premier mass spectrometer (Waters Corporation, Manchester, UK). Peptides were trapped and desalted prior to reverse phase separation using a Symmetry C18 5 *µ*m, 5 mm×300 *µ*m pre-column. Peptides were then separated prior to mass spectral analysis using a 10 cm×75 µm C18 reverse phase analytical column. Mass accuracy was maintained during the run using a lock spray of the peptide glu-fibrinopeptide B delivered through the auxiliary pump of the NanoAcquity at a concentration 200 fmol/µl and at a flow rate 500 nl/min. The LC-MS^E^ method acquires precursor and product ion data on all charge-states of an eluting peptide across its entire chromatographic peak width, providing more comprehensive precursor and product ion spectra. Peptides were analyzed in positive ion mode using a Q-ToF Premier mass spectrometer that was operated in v-mode with the resolving power of 10 000 fwhm. Prior to analyses, the ToF analyzer was calibrated using the doubly charge of glu-fibrinopeptide B (785.8426 m/z). Post calibration data files were corrected using the doubly charged precursor ion of glu-fibrinopeptide B (785.8426 m/z) with a sample frequency of 30 s. Accurate mass LC-MS data were collected in a data-independent and alternating low and high collision energy mode. The spectral acquisition time in each mode was 1 s with a 0.15 s interscan delay. In low energy MS mode, data were collected at constant collision energy of 10 eV. In MS^E^ mode, collision energy was ramped from 15 to 35 eV during each 1 s data collection cycle.

### Data processing and Database searching

ProteinLynx GlobalServer (PLGS) version 2.4 was used to process all data acquired. Protein identifications were obtained by searching the Swiss-Prot database. Protein identification from the low/high collision spectra for each sample was processed using an approach in which more than three fragment ions per peptide, seven fragment ions per protein and more than two peptides per protein, had to be matched. Since single-peptide protein identifications are more likely to represent false-positive data points, all proteins with greater than two peptides identified with less than 4% false discovery rate were considered as real hits. Carbamidomethylation was set as fixed modification and oxidation of methionine and N-acetyl terminal as variable modifications and no more than one miss cleavage was allowed. The ion detection, clustering, and normalization were processed using PLGS as described earlier [21].

### Ingenuity Pathway Analysis

A combined list of 37 proteins obtained from the proteomics analysis of tamoxifen, estrogen and ethanol treatments were recognized by the Ingenuity Pathway Analysis software (Ingenuity® Systems, www.ingenuity.com) and used to build networks and identify pathways based on data mining and connectivity into the set of these proteins providing an integrative view of the results.

### Western blot analysis

Total cell extracts were prepared by incubation of 10^6^ cells for 15 min on ice in 100 µl of lysis buffer [300 mM NaCl, 50 mM Tris pH 7.4, 1% Triton X-100 and protease inhibitors]. After clarification of the samples by centrifugation at 20 000 *g*, the supernatant was recovered. The protein concentration of the cell extracts and EVs were determined using Bradford protein assay (Bio-Rad, Hercules, CA). SDS-samples were incubated for 5 min at 37°C, 65°C and 95°C and separated on 4–12% pre-casted acrylamide gels (Invitrogen, Carlsbad, CA). Proteins were transferred to PVDF membranes and blocked overnight (5% milk and 0.05% Tween-20 in PBS), primary antibody was added for 1 hour, followed by PBS washing and incubation with the corresponding secondary HRP-conjugated antibody. All proteins were detected under non-reducing conditions. Chemoluminiscent detection of proteins was performed using ECL Plus reagent (Amersham).

### Nanoparticle Tracking Analysis (NTA)

Size distribution within EV preparations was analyzed by measuring the rate of Brownian motion using a NanoSight LM10 system, which is equipped with a fast video capture and particle-tracking software (NanoSight, Amesbury, U.K.).

### Internalization assay

M1, SK-Hep1, U2OS, SH-SY5Y and BXPC-3 cells were cultured in complete medium [DMEM containing 10% FBS, 4 mM glutamine and penicillin/streptomycin]. After plating 50 000 of each cell line/well on coverslips into 24-multiwell plates, they were left to grow for 16 hours. Then, the medium was changed to EV-depleted medium (the serum was centrifuged at 100,000 g for 16 hours to remove EVs before its use (Figure S1), and 100 µg/mL of EVs obtained from MDA-MB-468 cell cultures were added. After incubation for 24 hours, cells were washed in PBS three times and fixed in 2% formaldehyde-PBS solution for immunoassaying. Subsequently, coverslips were stained with anti-CD133 according to standard procedures. Finally, coverslips were mounted on DAPI containing Fluoromount G and analyzed under a 63X objective on a Leica TCS SP multiphoton confocal microscope.

### Stem cell and EMT marker assessment

MCF-7 or U2OS cells were cultured in complete medium [DMEM containing 10% FBS, 4 mM glutamine and penicillin/streptomycin]. After plating 50 000 cells/well in 24-well format, they were left to grow for 16 hours. The medium was then changed to EV-depleted medium and 50 µg/mL of EVs from MDA-MB-468 cells were added. After 48 hours of incubation, RNA was extracted using RNA Micro kit (Qiagen). Real-time PCR was performed on a 7300 Real-Time PCR System (Applied Biosystems), using the iTaq™ SYBR® Green Supermix with ROX (BioRad). 36B4 was used as a reference transcript for normalization. The sequences of the primers are the following: 36B4 FWD 5′ GTGTTCGACAATGGCAGCAT 3′, REV 5′ GACACCCTCCAGGAAGCGA 3′; OCT4 FWD 5′ GACAACAATGAAAATCTTCAGGAG 3′, REV 5′ CTG GCGCCGGTTACAGAACCA 3′; NANOG FWD 5′ CAGCTGTGTGTACTCAATGATAGATTT 3′, REV 5′ ACACCATTGCTATTCTTCGGCCAGTTG 3′, ZEB1 FWD 5′ AAGAATTCACAGTGGAGAGAAGCCA 3′, REV 5′ CGTTTCTTGCAGTTTGGGCATT 3′; SNAIL FWD 5′ CCTCCCTGTCAGATGAGGAC 3′, REV 5′ CCAGGCTGAGGTATTCCTTG 3′. The results are presented as fold change calculated with 2^−ΔΔCt^ method.

### Mammosphere formation assay

MCF-7 cells were grown in complete medium [DMEM containing 10% FBS, Glutamax and penicillin/streptomycin]. 20 000 cells/well were plated into 24-well plates. Next day, cells were treated with 25 or 50 µg/mL of EVs for 48 hours. Following exposure to EVs, cells were cultured in suspension at 10,000 cells/well in 6-well plates, and allowed to form mammospheres for 6 days before analysis.

### Electron microscopy

Cryo-electron microscopy of EVs was performed as previously described [20]. Briefly, a 4 µl droplet of the vesicle suspension was applied to a 200 mesh *R 2/2 Quantifoil*® holey-carbon grid. Excess of solution is removed with Whatman paper and the grid is rapidly plunged into liquid ethane and transferred under liquid nitrogen into the microscope using a side entry nitrogen-cooled *Gatan 914* cryoholder. Sample analysis was carried out under a *JEOL JEM 2200F* (Cs = 1.4 mm) transmission cryoelectron microscope, with an acceleration voltage of 200 kV and defocus ranging from −1.2 to −2.5 µm, accurately determined by using enhanced power spectra. Images were recorded under low dose conditions (10 electrons per A^2^) with a *2k*×*2k Gatan Utrascan™ 1000* CCD camera.

## Results

### Secretion of extracellular vesicles by primary human mammospheres

**Figure 1:**
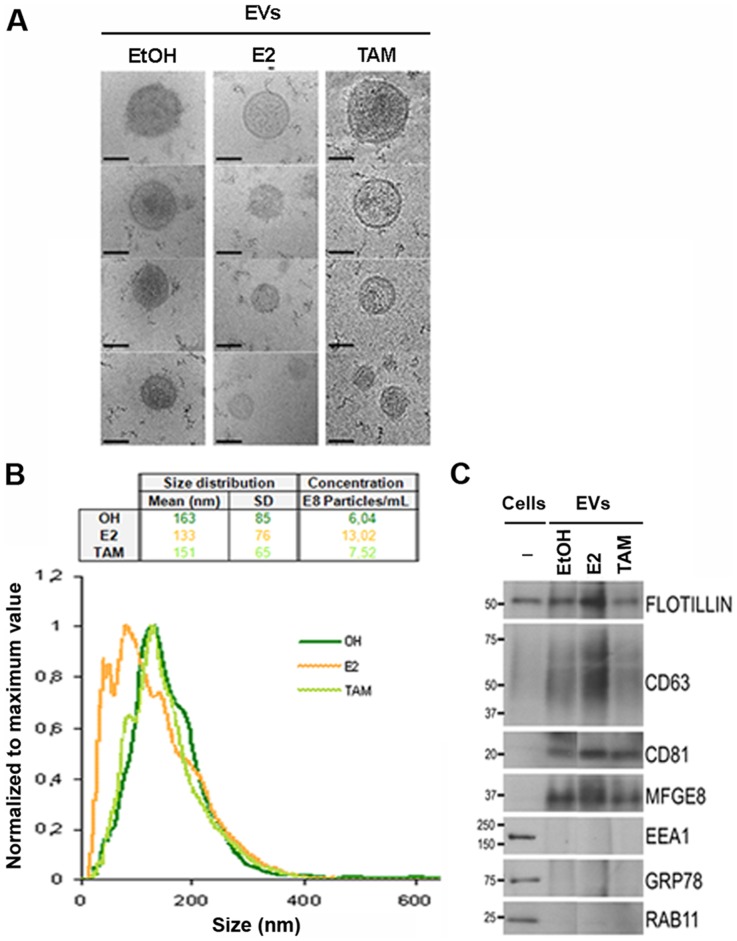
Characterization and comparison of primary mammosphere-derived EVs cultured with or without hormone treatments. (A) Representative cryo-electron micrographs (Bar, 100 nm). (B) Normalized representation of size distribution by NTA analysis of EVs in one primary breast epithelial cell preparation. Mean, SD and particle concentration values are indicated in the table (EtOH in green, E2 in orange and TAM in light green). (C) Western blot analysis of cell extracts and EVs derived from mammospheres treated with ethanol (EtOH), estrogen (E2) or tamoxifen (TAM). Antibodies against exosomes (Flotillin-1, CD63, CD81 and MFGE8), early (EEA1) and recycling (Rab11) endosomes, or endoplasmic reticulum (Grp78) protein markers were assayed. The molecular mass (kDa) for each protein is indicated.

Primary human normal breast epithelial cells were cultured in suspension to enrich for cells with properties of stem/progenitor cells. In order to investigate the potential influences of hormones on EVs secretion, we analyzed the secreted material in mammosphere cultures treated with estrogen, tamoxifen or the carrier ethanol. Cryo-electron microscopy showed that this material contained round-shaped vesicles with a limiting membrane clearly defined (Figure 1A). Although, the secretion of vesicles was observed in all cases, according to NTA analysis the size distribution showed some differences due to hormone treatment (Figure 1B), especially by addition of estrogen, which led to increased secretion of smaller size vesicles. Western blot analysis confirmed the enrichment of protein markers for exosomes, namely Flotillin-1, CD63, CD81 and MFGE8, with respect to the original cell extracts (Figure 1C). Importantly, while in the total cell extracts the presence of endoplasmic reticulum (Grp78) or early and recycling endosomes (EEA1 and RAB11) was clearly detected, these markers were undetectable in the same amount of protein prepared from the secreted material (Figure 1C), indicating that these vesicles were not a consequence of cell lysis or other organelle contamination and that EV secretion is an active and controlled process. Furthermore, we run parallel experiments with and without cells (only medium) and the material obtained in the latter case after the EV purification procedure was undetectable (Figure S1B). In addition, this level of characterization showed that both estrogen or tamoxifen treatments affected the expression levels of several proteins associated with EVs that were secreted by the breast epithelial cells, such as Flotillin-1 and CD63 (Figure 1C). Taken together, these data indicate that primary human mammospheres secrete EVs into the extracellular environment, and that their size and protein content reflect cellular responses to hormone treatment.

### Tamoxifen regulates the protein content of mammosphere-derived EVs

**Figure 2:**
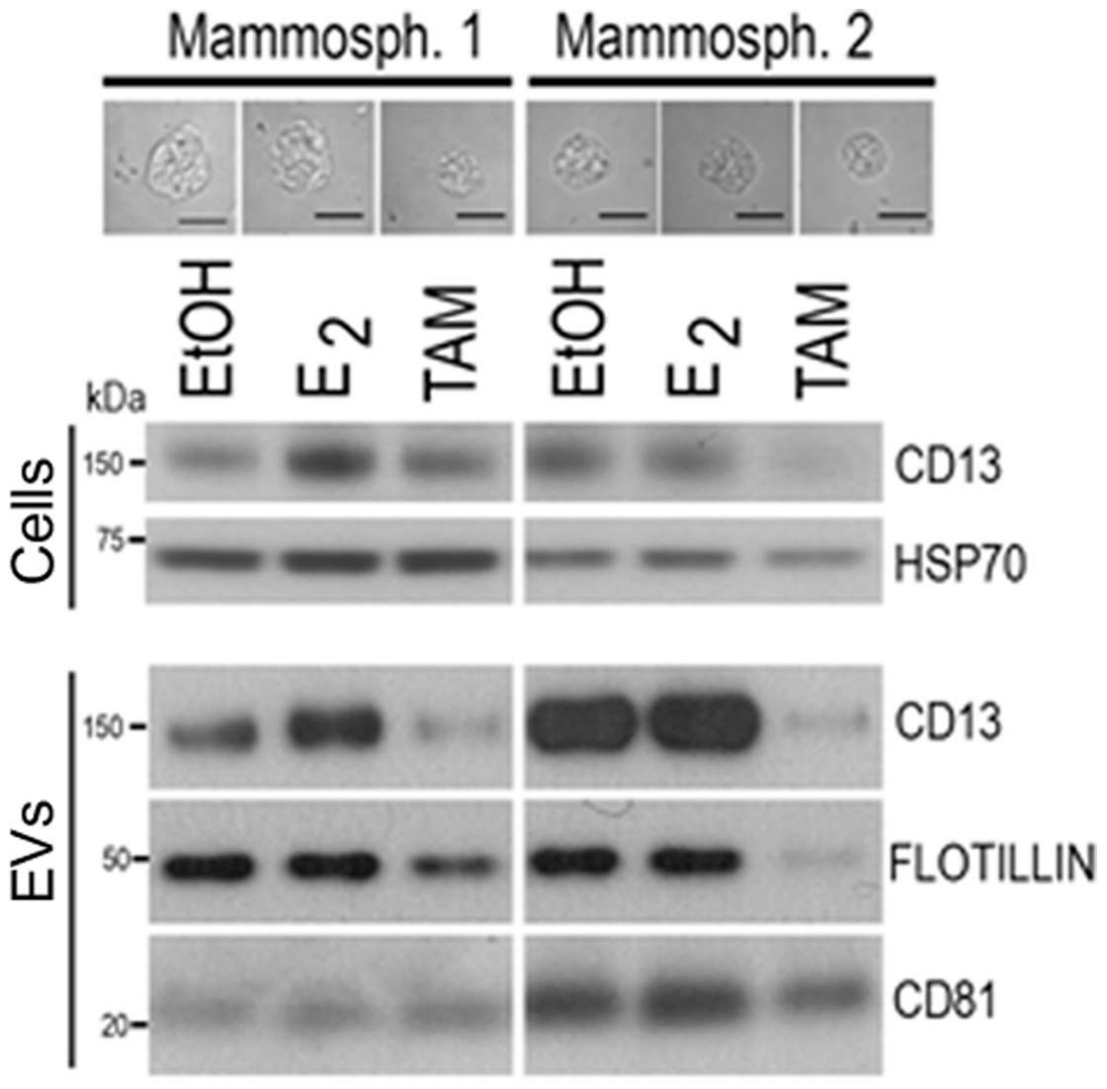
EV content is modulated by hormone treatment. Western blot analysis of total cell extracts and EVs of two independent human primary mammosphere preparations cultured in the presence of ethanol (EtOH), estrogen (E2) or tamoxifen (TAM). Protein expression was analyzed by immunoblotting with antibodies against CD13, Flotillin-1 and CD81 proteins. The molecular mass (kDa) for each protein is indicated. Representative micrographs of mammospheres formed under the different conditions are also shown. *Bar, 50* µ*m*.

Our findings suggest that EVs could be used as non-invasive indicators for monitoring cellular response to hormone treatment. To provide further evidence to support this possibility, we analyzed EVs secreted from two independent mammosphere preparations (matched for similar age and endocrine background). Mammosphere formation was confirmed in all cases under the light microscope and, as expected, tamoxifen treatment led to mammospheres of smaller size (Figure 2), in agreement with our previous report [19]. Due to the low amount of EVs secreted, we focused our analysis on proteins known to be found in these vesicles and for which well-characterized antibodies are available, namely CD13, Flotillin-1, and CD81. These three proteins were detected in all EV preparations, and the expression levels of Flotillin-1, and particularly CD13, were reduced in EVs secreted by tamoxifen-treated mammospheres (Figure 2). These findings further support that the composition of the EVs can reflect changes that have occurred in the cells of origin in response to hormone treatment.

### Proteomics of extracellular vesicles derived from primary mammospheres

**Figure 3:**
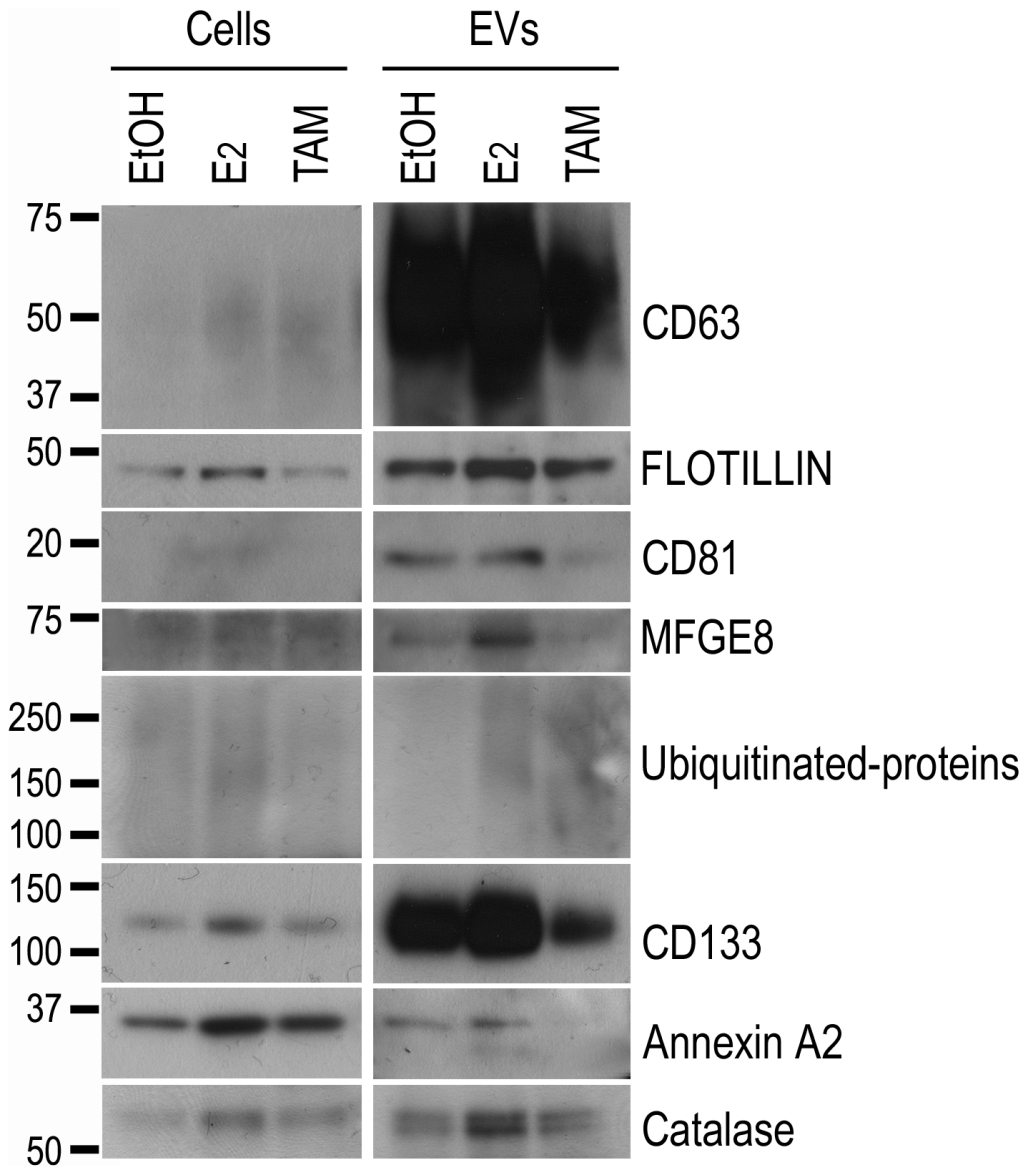
Validation of proteomics profiling of EVs from primary human mammospheres by immunoblotting. Western blot analysis of protein extracts obtained from cells (left) or EVs (right) secreted by mammospheres cultured in the presence of ethanol (EtOH), estrogen (E2) or tamoxifen (TAM). In addition to EV protein markers (Flotillin-1, CD63, CD81 and MFGE8) used as positive controls, the presence of proteins detected in the proteomic analysis, including prominin1/CD133, annexin A2 and catalase, were evaluated. Three different tissue samples were examined and one representative example is shown. The molecular mass (kDa) for each protein is indicated.

To analyze the protein content of the mammosphere-derived EVs in more detail we used liquid chromatography mass spectrometry-based proteomics. Equal amounts of EVs obtained from mammospheres cultured in the presence of ethanol (control), estrogen or tamoxifen were processed for proteomics analysis. The base peak intensity (BPI) observed for the three samples (ethanol, estrogen or tamoxifen) was very similar, reflecting that the same amount of protein was loaded for the three samples (Figure S2). Among the different proteins identified (Table 1), the majority of them have previously been found in EVs, according to Vesiclepedia and Exocarta databases [22], further supporting the novel observation that primary mammospheres secrete EVs. Only 3 proteins, ERGI2, RUFY2 and NFS1, were not found in the mentioned databases. Some of the proteins were detected independently of the treatment. However, other proteins, such as annexin A2 and some cytosolic enzymes (e.g. HCD2, DHE3, TPIS, ALDOB) were detected only in some of the conditions tested (Table 1). Taking into account that the same amount of EV protein from each of the treatments was injected, the differential expression observed for some of the proteins suggests that mammosphere-derived EVs are sensitive to environmental changes, such as hormone treatment, in agreement with the findings described above. To support the proteomics results, Western blot analysis confirmed that expression levels of CD133, annexin 2, catalase and ubiquitinated-proteins of high molecular weight were altered in EVs isolated from human breast epithelial cell suspension cultures treated with estrogen or tamoxifen (Figure 3).

**Table 1:** Proteomic profiling of Evs secreted by breast primary human mammospheres.

OH^a^	E2^a^	TX^a^	PROTEIN ID	PROTEIN NAME	IPA^b^
					Can	Prol	Dev
				*Membrane-associated proteins*			
36	64	59	TRFL_HUMAN	Lactotransferrin	x	x	x
	9		MFGM_HUMAN	Lactadherin/Milk fat globule-EGF factor 8	x	x	x
	13	8	ANXA2_HUMAN	Annexin A2	x	x	x
	23		PROM1_HUMAN	Prominin-1/CD133	x		
	17		AMPN_HUMAN	Aminopeptidase N/CD13	x	x	x
	2		ERGI2_HUMAN	ER Golgi intermediate compartment protein 2			
	6		RAB15_HUMAN	Ras related protein Rab 15			x
	8		RUFY2_HUMAN	RUN and FYVE domain containing protein 2	x		
	11		HS90A_HUMAN	Heat shock protein HSP 90-alpha	x	x	x
		3	BAK_HUMAN	Bcl 2 homologous antagonist killer	x	x	x
				*Enzymes*			
3	12	8	G3P_HUMAN	Glyceraldehyde-3-phosphate dehydrogenase	x	x	x
8	11		ALDOB_HUMAN	Fructose-bisphosphate aldolase B	x	x	
	8		ALDOC_HUMAN	Fructose bisphosphate aldolase C	x	x	
2	3		TPIS_HUMAN	Triosephosphate isomerase	x		
	6		KPYM_HUMAN	Pyruvate kinase isozymes M1/M2	x	x	
	10	13	DHE3_HUMAN	Glutamate dehydrogenase 1, mitochondrial	x	x	x
	4		HCD2_HUMAN	3-hydroxyacyl-CoA dehydrogenase type-2	x	x	
2	12	8	SAHH_HUMAN	Adenosylhomocysteinase		x	x
	11	4	AL1A1_HUMAN	Retinal dehydrogenase 1	x	x	x
	8		FTHFD_HUMAN	10 formyltetrahydrofolate dehydrogenase	x		
		7	DPYS_HUMAN	Dihydropyrimidinase			
	4		ACADS_HUMAN	Short-chain acyl-CoA dehydrogenase			
	6		THIM_HUMAN	3-ketoacyl-CoA thiolase, mitochondrial			
		3	NFS1_HUMAN	Cysteine desulfurase mitochondrial		x	
	7		CATA_HUMAN	Catalase	x	x	x
				*Miscellaneous*			
11	20	17	ALBU_HUMAN	Serum albumin	x	x	x
3	9	6	UBIQ_HUMAN	Ubiquitin	x		
	5		PSD10_HUMAN	26S proteasome non-ATPase reg sub10		x	
		13	ACTA_HUMAN	Actin, aortic smooth muscle	x		
12	28		ACTB_HUMAN	Actin, cytoplasmic 1	x	x	x
	26		ACTG_HUMAN	Actin cytoplasmic 2	x	x	x
	16		CO6A1_HUMAN	Collagen alpha-1(VI) chain		x	x
	10		CO6A2_HUMAN	Collagen alpha-2(VI) chain		x	x
		4	TRY6_HUMAN	Putative trypsin 6			
3			H2A1B_HUMAN	Histone H2A type 1 B			
	20	6	H2AX_HUMAN	Histone H2A x		x	x
6	10	15	H2B1K_HUMAN	Histone H2B type 1 K	x		
21			H31_HUMAN	Histone H3.1			
	17	36	H33_HUMAN	Histone H3.3			
12	15	10	H4_HUMAN	Histone H4			
11	9	8	H13_HUMAN	Histone H1.3		x	
6	4	4	H14_HUMAN	Histone H1.4		x	

^a^ Number of peptides identified in the mass spectrometry analysis.

^b^ Functional categorization using Ingenuity Pathway Analysis software. The functional categories more represented in the proteomics analysis were Cancer (Can), Proliferation (Prol) (including cellular growth, cell cycle and cell death) and Development (Dev) (including cell signaling, cellular assembly and organization, tissue remodeling and maintenance).

**Figure 4:**
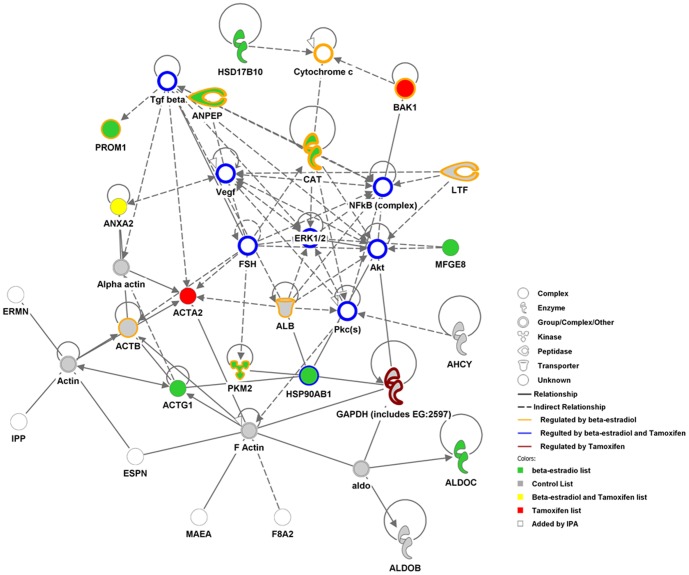
Cancer-related networks detected by the data mining IPA software. A list of 37 proteins was recognized by ingenuity pathway analysis software to build a connectivity network. This network was highly associated to cancer (*p*-value = 5.9E-12) and integrates the data from the list of proteins identified in control (grey-filled circles), in estrogen (green-filled circles), in tamoxifen (red-filled circles) or in both estrogen and tamoxifen (yellow-filled circles) treatments. Some molecules (unfilled) were added by IPA software to complete the pathway. The proteins known to be regulated by estrogen (surrounded by an orange outline), by tamoxifen (surrounded by violet outline) or both (surrounded by blue outline) are also indicated.

In order to obtain an integrative view of the proteins identified in the proteomics analysis and to detect possible pathways in which they could be involved we used specialized data mining software (Ingenuity Pathway Analysis, IPA). Out of the 42 proteins identified, the software recognized 37 and, remarkably, 29 of them have been categorized as cancer-related molecules (Table 1, indicated as “Cancer”), suggesting a possible involvement of these EVs in cancer progression and metastasis as recently demonstrated for melanoma [23]. Indeed, the highest score connectivity network that the IPA software was able to create with the data set was a cancer-related network (illustrated in Figure 4). In addition, many of the proteins, including prominin-1 (CD133), aminopeptidase-N, lactotransferin, BAK1, HSP90, β-actin and the enzymes GAPDH, pyruvate kinase M2 and catalase, were previously reported to be regulated by estrogen and/or tamoxifen, according to the information in the IPA database.

### Cancer cell-derived EVs alter expression of stem cell and EMT factors in recipient cells

**Figure 5:**
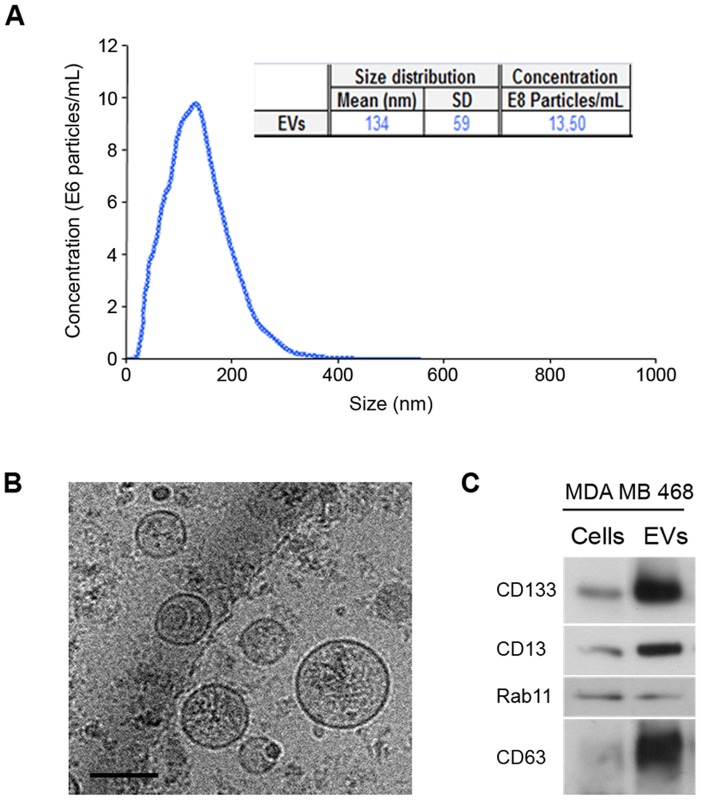
Characterization of EVs secreted by MDA-MB-468 mammospheres. (A) NTA analysis indicating mean, standard deviation and concentration of the particles present in the preparation. (B) Representative cryo-electron micrographs (*Bar, 100 nm*). (C) Western blot analysis of protein extracts prepared from cells or from EVs using antibodies against indicated proteins.

Breast cancer mortality is mainly due to the spreading of tumor cells to secondary sites (tumor metastasis) in the body. Given the role of stem cells in tumorigenesis and invasiveness, we analyzed the capacity of the mammosphere-derived EVs to participate in the transfer of signals between breast cancer cells and to contribute to their invasion capacity. To this end, we examined the EVs in mammospheres obtained from MDA-MB-468 breast cancer cells, which are more metastatic than ER-positive cells. Analysis of particle size profile (Figure 5A), electron microscopy (Figure 5B) and Western blot (Figure 5C) showed that mammospheres isolated from MDA-MB-468 cells also secreted EVs containing exosomal markers CD63 and Flotillin-1, as well as CD133 and CD13 proteins, as previously observed with mammospheres from primary breast cells.

**Figure 6:**
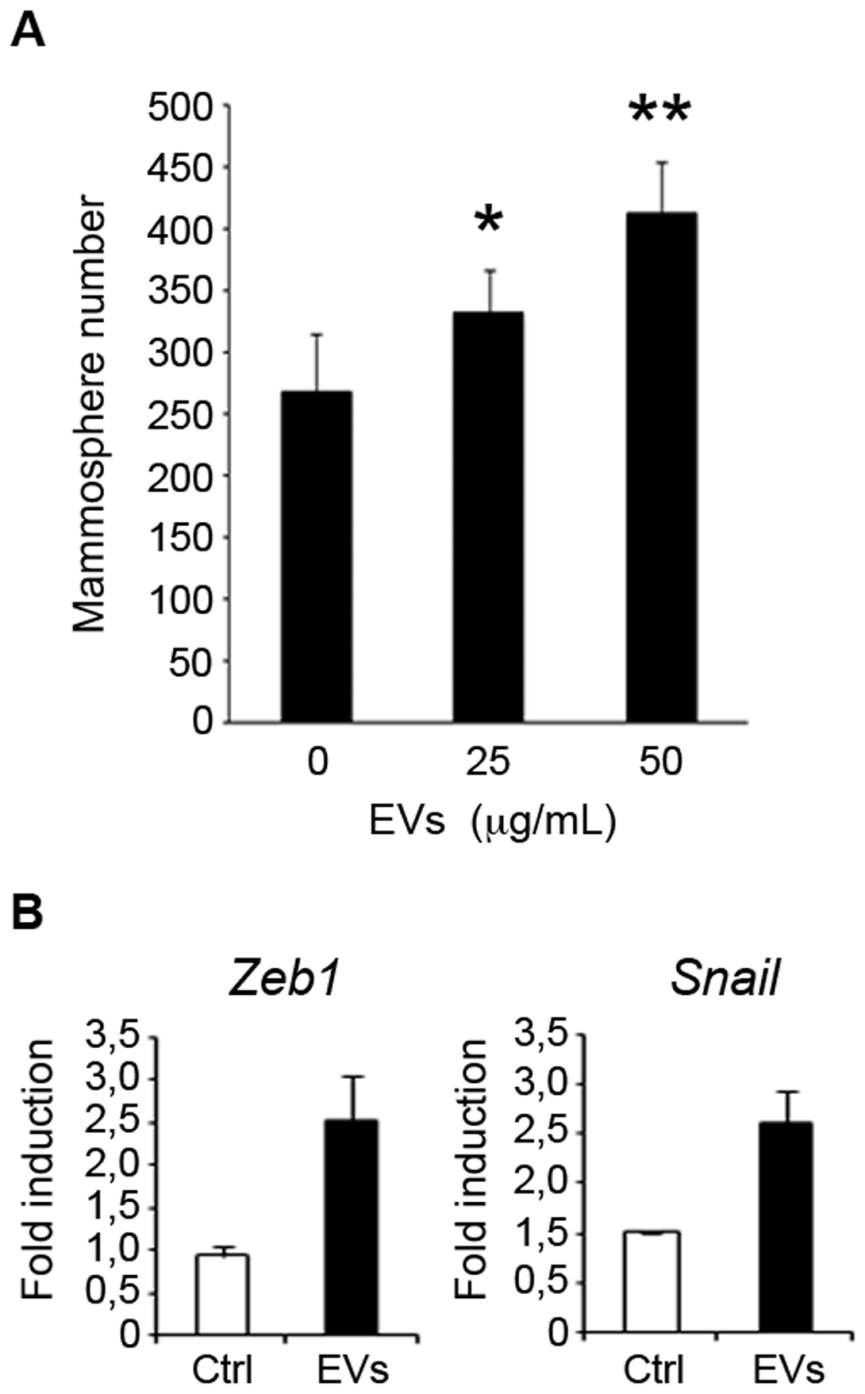
Effect of EVs secreted by MDA-MB-468 mammospheres on MCF7 cells. (A) MCF-7 cells were incubated with 0, 25 and 50 µg/mL of MDA-MB-468 EVs and the number of mammospheres formed after 7-days was counted [data are mean ±SD; *n* = 3, *p<0.05, **p<0.01, relative to the values in the control]. (B) Quantitative polymerase chain reaction analysis was conducted to examine the expression of the factors *Zeb1* and *Snaill* [data are mean ±SD; *n* = 3, *p*<0.05 and 0.05 respectively, relative to the values in the control].

To get insight into the function of EVs, ER-positive MCF-7 breast cancer cells were incubated with EVs from ER-negative MDA-MB-468-derived mammospheres. Interestingly, MCF-7 cells exposed to EVs derived from more aggressive cells exhibited a significantly increased capacity to form mammospheres and this ability was dose dependent (Figure 6A). Analysis of the EV-treated cells revealed the induction of genes encoding Zeb1 and Snail proteins, which have been implicated in the epithelial mesenchymal transition (EMT) (Figure 6B). These findings suggest that EVs from metastatic cells can affect the behavior of less aggressive neighboring cells.

**Figure 7:**
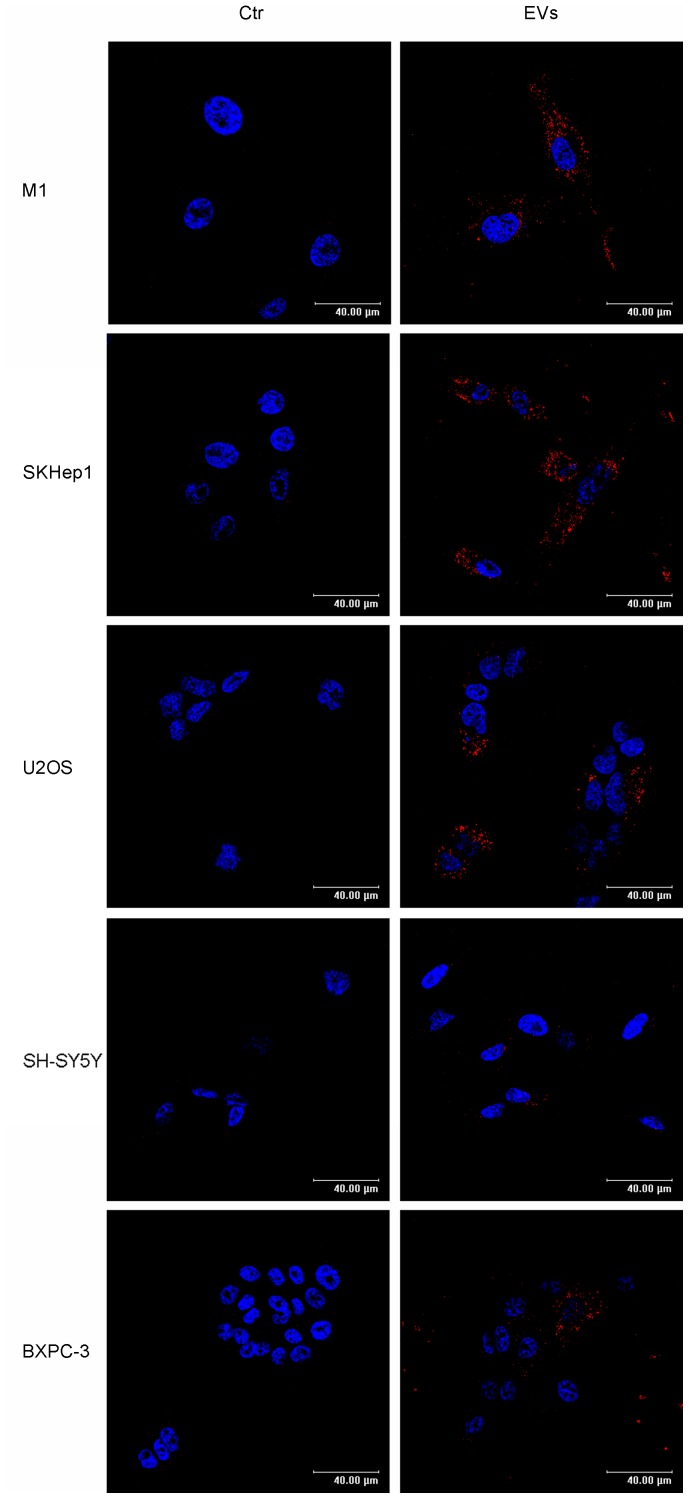
Uptake of EVs by cell lines of different tissue origin as putative metastatic destinations. Left panels represent images of M1 (fibroblast), SK-Hep1 (endothelial, liver adenocarcinoma), U2SO (osteosarcoma), SH-SY5Y (neuroblastoma) and BXPC3 (pancreatic adenocarcinoma) human acceptor cells under control conditions. Right panels are individual frames showing the internalization of CD133-positive EVs from MDA-MB-468 into the acceptor cells (*Bar, 40 µm*).

**Figure 8:**
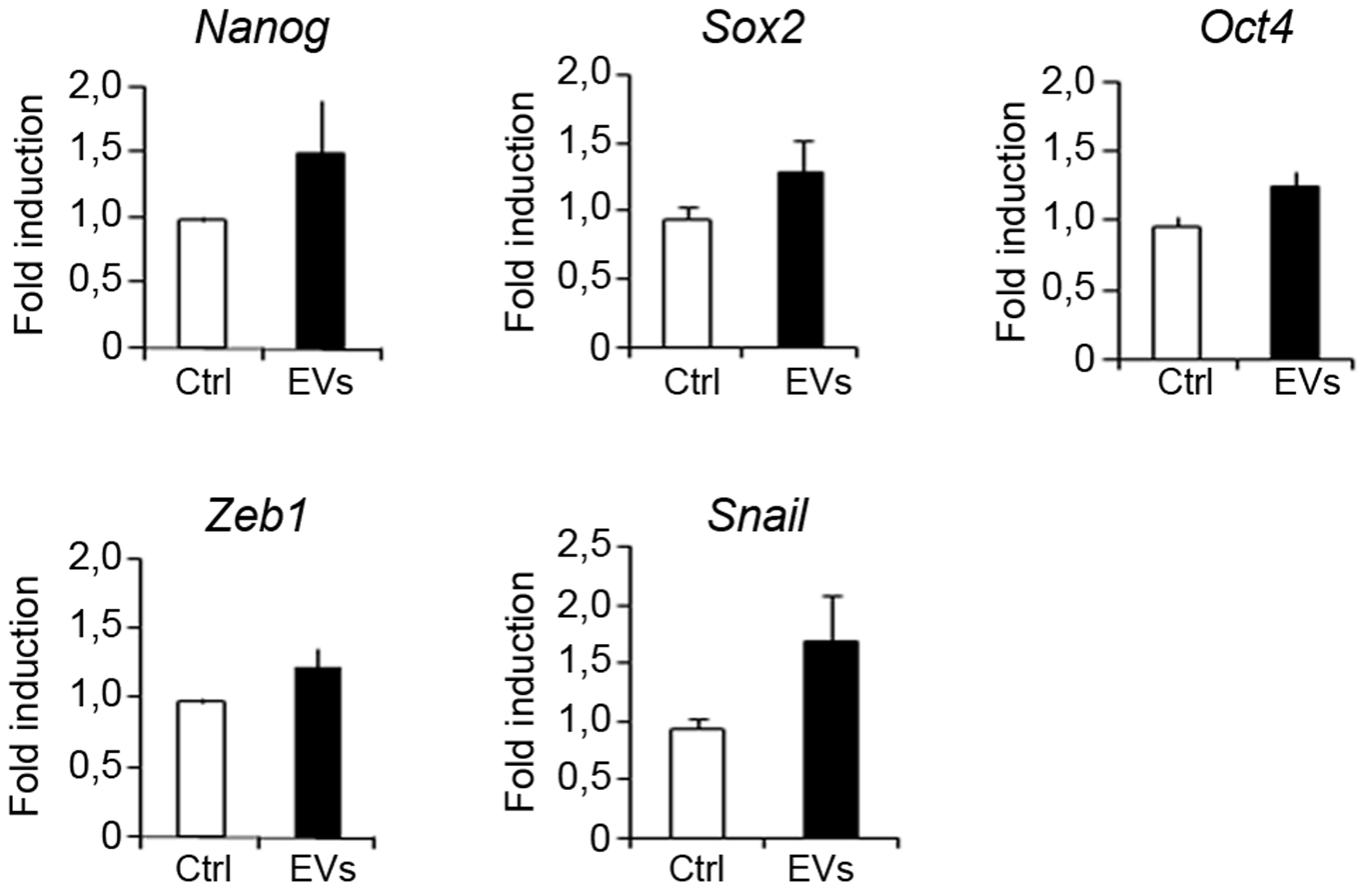
Effect of EVs from MDA-MB-468 on stem cell and EMT markers in U2OS cells. U2OS cells were incubated with 0 or 50 µg/mL of MDA-MB-468-derived EVs and quantitative polymerase chain reaction analysis was conducted to examine the expression of the transcription factors *Nanog*, *Oct-4*, *Sox2*, *Zeb1* and *Snaill* involved in development and maintenance of stem cells [data are mean ±SD; *n* = 3, *p*<0.05, 0.05, 0.01, 0.05 and 0.01, respectively, relative to the values in the control].

Cancer stem cells are known to be involved in tumor propensity to colonize specific organs. For example, breast cancer cells metastasize preferentially to bone, lungs, liver and brain. Previously, EVs have been shown to confer some of the transformed characteristics of the donor cancer cells onto normal cells [14]. To examine the potential of EVs to transfer information to cells of different tissue origins we isolated EVs shed by MDA-MB-468 cells cultured as mammospheres. To conduct uptake experiments, we took advantage of the fact that these vesicles are enriched with Prominin1/CD133, a stem cell marker that was also detected by proteomics in EVs from primary human normal epithelial cells. EVs were captured by cells of different origins, as suggested by the presence of CD133 in different recipient cells, including M1 (human fibroblastoid cell line), SK-HEP-1 (human hepatic endothelial cells), U2OS (human osteosarcoma cell line), and less efficiently by SH-SY5Y (human neuroblastoma cell line) and BXPC3 (human primary pancreatic adenocarcinoma cells) (Figure 7). Moreover, incubation of U2OS cells with EVs increased the expression of stem cell markers *Nanog*, *Oct4* and *Sox2*, as well as the EMT markers *Zeb1* and *Snail* (Figure 8). These findings suggest that the transfer of material from metastatic aggressive breast cancer cells is capable of providing neighboring and long distance recipient cancer cells with an aggressive phenotype.

## Discussion

In this study we provide, for the first time, structural and biochemical evidence showing that normal primary mammospheres are able to secrete EVs to the extracellular environment. Furthermore, we demonstrate that the EV protein content is affected by hormone treatment. Finally, EVs secreted by breast cancer cells can confer a more aggressive molecular phenotype to recipient cells.

The proteomic analysis of the vesicles identified cytosolic proteins (e.g. Hsp90A and G3P), cytoskeleton-associated proteins (e.g. actins), and several histones. In addition, ubiquitin was also found in our proteomic analysis, suggesting the presence of ubiquitinated proteins. These findings are consistent with the characterization of EVs from other sources [8].

An interesting point revealed by our study is that the protein content of mammosphere-derived EVs is affected by exposure to estrogen and tamoxifen. For example, lactadherin/MFG-E8 is a glycoprotein found as a predominant component of the mammary fat globule that is released into milk and is involved in preventing infections in breast-fed infants [24]. In this study, we observe that MFG-E8 protein expression in EVs is increased in response to estrogen and reduced in response to tamoxifen. Consistent with this finding, MFG-E8 expressed by mammary gland epithelial cells can bind to α_v_β_3/5_ integrin receptor thereby activating MAPK and, as a consequence, triggering cell proliferation and duct outgrowth [25]. Furthermore, MFG-E8 is implicated in vascularization in both healthy and tumor cells [26], [27], and is expressed and often up-regulated on the surface of breast carcinoma cells [28]. We also find that Flotillin-1 protein expression is reduced by tamoxifen treatment. Flotillin-1 is a raft protein that can co-localize with ER in lipid rafts from MCF-7 breast cancer cells, leading to modulation of cell growth [29]. Lipid raft structure is known to play an important role in various cellular processes, including signal transduction, and breast cancer cell invasion [30].

Another hormone-regulated protein identified in this study is aminopeptidase N (APN/CD13), a transmembrane Zn^2+^-dependent ectopeptidase that cleaves N-terminal neutral amino acids of various peptides and proteins, and is implicated in various processes, including cell proliferation, tumor invasion and angiogenesis [31], [32], with high expression correlating with malignancy [33]. Some controversy exists in the literature regarding the hormonal regulation of APN/CD13, likely due to cell-type specific differences [34], [35], [36]. We observe that APN/CD13 expression in EVs is increased by estrogen and strongly reduced by tamoxifen treatment in breast epithelial sphere cultures. Interestingly, CD13 expression is induced during differentiation and is expressed at lower levels in side population (SP) cells than in non-SP cells in human endometrium [37]. The SP phenotype has been associated with the presence of a stem cell subpopulation in many different cell types, including the mammary gland [18], [38]. Consistent with this, we have previously shown that estrogen promotes differentiation of normal breast stem cells leading to a reduction of the stem cell subpopulation [19].

By using specialized data mining software, a cancer-related network could be built that contained most of proteins identified in mammosphere-derived EVs proteomics, suggesting a potential role of these EVs in the development of cancer. This study represents a novel report of the secretion of EVs from primary breast suspension cultures, enriched for stem/progenitor cells. Taking these findings together, the presence of these proteins in mammosphere-derived EVs could suggest a possible role of EVs in immune function and/or tumor progression to a more aggressive phenotype. The mechanisms of cell-to-cell communication are not well understood, but it may have significant consequences in cancer progression. In particular, recent developments in the field suggest that cancer cell-derived EVs may induce transformation of normal recipient cells [14]. Interestingly, several studies have reported that the presence and frequency of a subpopulation of cancer stem cells has prognostic relevance, a high cancer stem content within the tumor correlates with aggressive poorly differentiated tumors [39], [40].

In this work, we showed that EVs from mammospheres enriched in cancer stem cell are able to increase the formation of MCF7 mammospheres in a dose dependent manner, which correlates with an increase in the gene expression levels of proteins involved in epithelial-mesenchymal transition. This finding supports a role of these vesicles in the malignant transformation of recipient cells even at a long distance from the primary tumor that could facilitate the establishment of metastasis in clear agreement with recent reports on EVs secreted by different cancer cells [14], [23], [41], [42]. Indeed, we have also shown the capture capacity of various cell lines related to organs where metastasis of breast cancer cells commonly occur including bone (U2OS), brain (SH-SY5Y), liver (SK-Hep1) and pancreas (BXPC-3). Remarkably, the osteosarcoma U2OS cells internalized significant amount of EVs and the incubation with EVs led to an increase in the expression of EMT genes *Zeb1* and *Snail* and of the stem cell markers Nanog, Oct4 and Sox2. These findings suggest that the vesicles can confer phenotypic traits to recipient cells similar to their cell of origin, and are consistent with the idea that the shedding of EVs by cancer cells may provide signals to the microenvironment to induce or maintain tumorigenesis. Our observations merit further research into the correlation between the presence of stem cells and EV secretion, and their potential as diagnostic and/or prognostic markers.

Much work remains to be done to examine the secretion of EVs by stem/progenitor and differentiated breast cells, and to compare it to that of cancer stem cells. However, the potential of EVs to be used as non-invasive diagnostic tools for detecting breast disease or to monitor response to endocrine therapy is an attractive prospect that warrants further investigation.

## Supporting Information

Figure S1 **Analysis of EV-depleted medium.** (A) Western blot analysis of CD63, Rab11, CD13 and CD133 levels in EVs obtained from MDA-MB-468 cells or EVs obtained as a result of EV-depletion of the medium used for the internalization assays. As positive control for the antibodies against the tested proteins (CD63, Rab11, CD13 and CD133) we have included in the analysis equal amount of protein obtained from EVs released by MDA-MB-468 mamospheres. Note that the antibodies do not cross react with any protein in the bovine EVs, probably due to the fact that the antibodies are species-specific. (B) NTA analysis of EVs purified from medium that has been incubated in parallel in the absence or presence of human primary mammospheres. Note that vesicles were undetectable in the media incubated in the absence of cells, indicating that the EVs purified from the media that was incubated with cells must had been released by the cells.
(TIF)

Figure S2 **Chromatograms with base peak intensity (BPI) in breast epithelial cells.** The chromatograms show the base peak intensity (BPI) in the three samples (ethanol, tamoxifen and estrogen treated) used to perform the proteomic analysis. Note: similar intensity was observed in all cases supporting the fact that similar amount of protein was loaded for the three samples. In addition, the number of masses detected was very similar as well (ethanol: 19969, estrogen: 17974 and tamoxifen: 20024 masses).
(TIF)

Materials and Methods S1 (DOC)
