# Supplementary figures and images for: Human Mammospheres Secrete Hormone-Regulated Active Extracellular Vesicles
Source: PLoS One. 2014 Jan 3;9(1):e83955. doi: 10.1371/journal.pone.0083955 (PMC3880284; doi:10.1371/journal.pone.0083955)

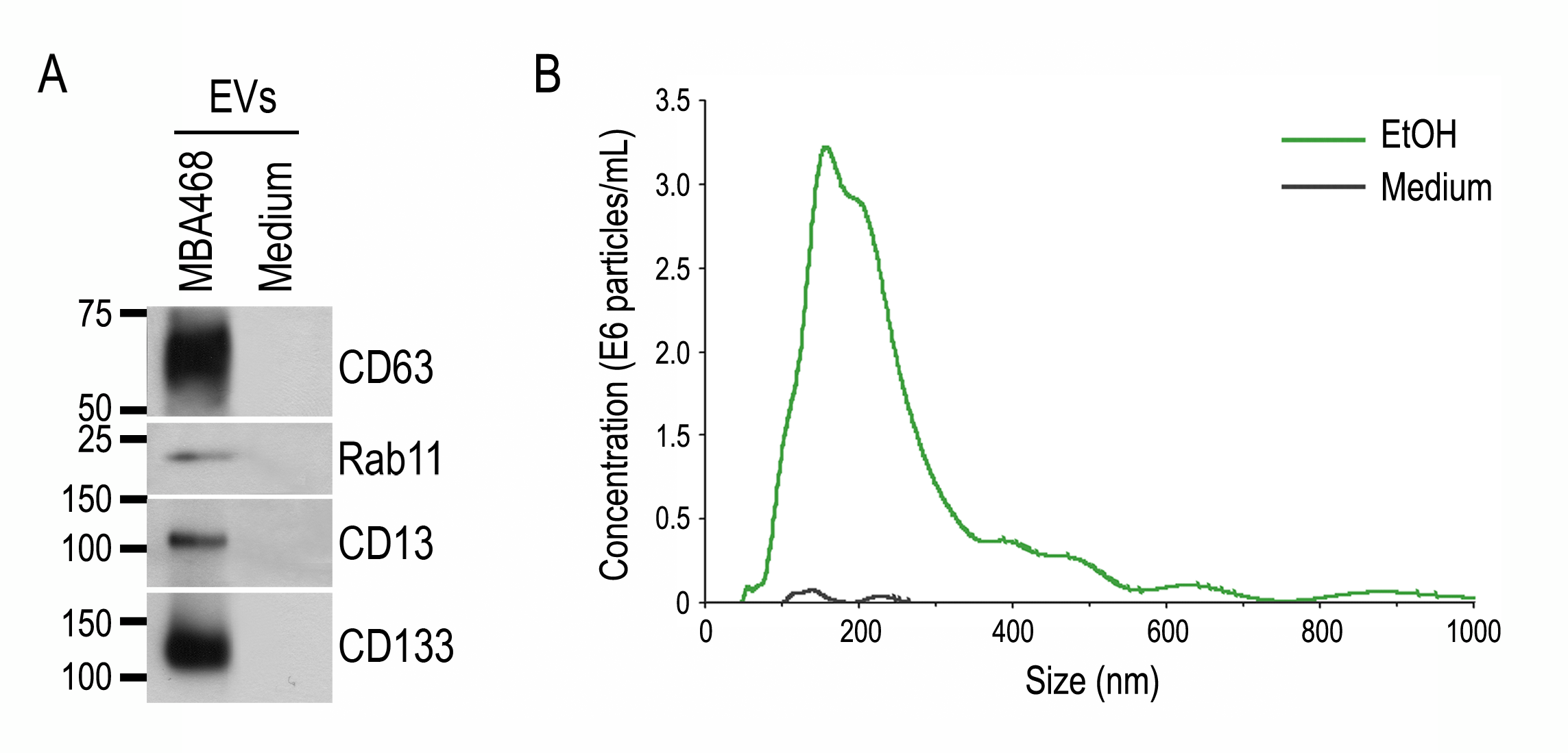

Supplement: Figure S1 — Analysis of EV-depleted medium. (A) Western blot analysis of CD63, Rab11, CD13 and CD133 levels in EVs obtained from MDA-MB-468 cells or EVs obtained as a result of EV-depletion of the medium used for the internalization assays. As positive control for the antibodies against the tested proteins (CD63, Rab11, CD13 and CD133) we have included in the analysis equal amount of protein obtained from EVs released by MDA-MB-468 mamospheres. Note that the antibodies do not cross react with any protein in the bovine EVs, probably due to the fact that the antibodies are species-specific. (B) NTA analysis of EVs purified from medium that has been incubated in parallel in the absence or presence of human primary mammospheres. Note that vesicles were undetectable in the media incubated in the absence of cells, indicating that the EVs purified from the media that was incubated with cells must had been released by the cells. (TIF) [file pone.0083955.s001.tif]

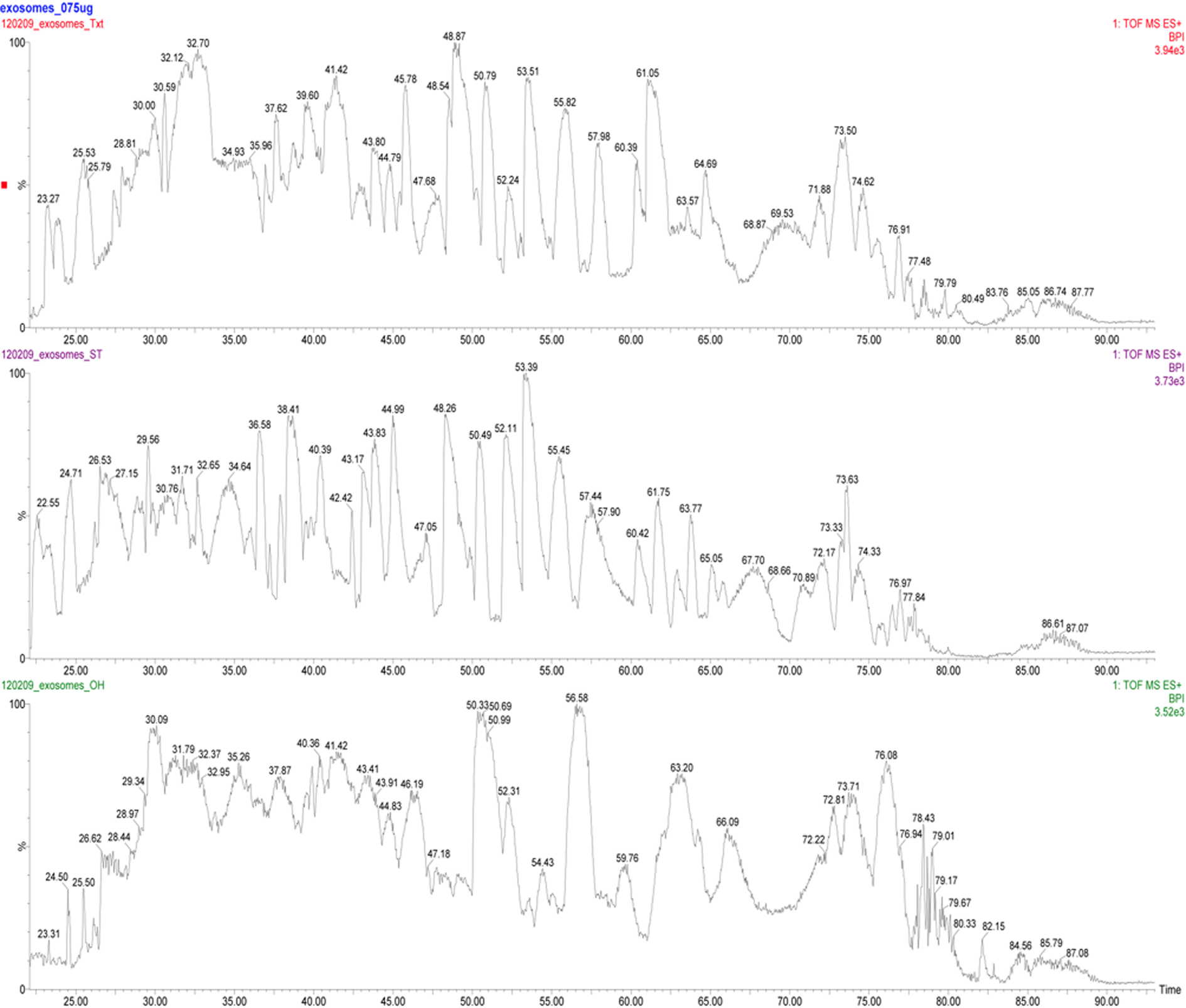

Supplement: Figure S2 — Chromatograms with base peak intensity (BPI) in breast epithelial cells. The chromatograms show the base peak intensity (BPI) in the three samples (ethanol, tamoxifen and estrogen treated) used to perform the proteomic analysis. Note: similar intensity was observed in all cases supporting the fact that similar amount of protein was loaded for the three samples. In addition, the number of masses detected was very similar as well (ethanol: 19969, estrogen: 17974 and tamoxifen: 20024 masses). (TIF) [file pone.0083955.s002.tif]
