# Supplementary material for: Human Mammospheres Secrete Hormone-Regulated Active Extracellular Vesicles
Source: PLoS One. 2014 Jan 3;9(1):e83955. doi: 10.1371/journal.pone.0083955 (PMC3880284; doi:10.1371/journal.pone.0083955)
Supplement: Materials and Methods S1 — (DOC) [file pone.0083955.s003.doc]

**Gonzalez, Piva et al., Supporting Information**

**Materials and methods**

Human tissue collection and primary mammosphere culture

Normal breast tissue was obtained from women undergoing reduction mammoplasties with no previous history of breast cancer. All patients provided written informed consent, and the local Hospital Research Committee and the “Ethics Committee of Clinical Investigation of Euskadi” approved the procedures. The breast tissue was immediately processed as previously described to yield a predominantly single cell suspension [24].

Breast epithelial cells were grown in suspension in phenol red free DMEM:F-12 medium with GlutaMAX, supplemented with B27, 20 ng/ml EGF, 20 ng/ml bFGF and 1% penicillin/streptomycin at 37°C in 5% CO_2_, as previously described [25]. Single cell suspensions were plated at a density of 50.000 cells/ml in 75 cm^2^ ultralow attachment flasks (Corning) and treated with ethanol, as control, 10^-8^M 17-β-estradiol (estrogen) or 10^-7^M 4-OH-tamoxifen (tamoxifen). After 7 days, mammospheres were collected by centrifugation at 400 *g* and MVs were isolated.

Production and isolation of extracellular microvesicles

For MV production, 60 ml of supernatant from primary human mammosphere cultures were collected by centrifugation and material secreted into the medium was purified as previously described [18]; briefly, culture supernatant was collected and centrifuged at 500 *g* for 10 min to remove cells. The resultant supernatant was subjected to filtration through 0.22 µm pore filters, followed by ultracentrifugation at 10.000 *g* and 100.000 *g* for 30 min and 60 min, respectively. The resulting pellets were washed with PBS and again ultracentrifuged at 100.000 *g* for 60 min. The final pellet of MVs was resuspended in 1/1000^th^ of the original volume of the culture supernatant, and stored at -80ºC. Total protein content of MV preparations was 13.50±2.93 μg (n=6), leading to an estimate of 4.51 pg/cell of MV production.

Liquid Chromatography-Mass Spectrometry (LC-MS^E^)

Proteins were identified by direct analysis of the reaction mixture described above. All analyses were performed in triplicate (500 ng of material in each injection) using a NanoAcquity UPLC and Q-ToF Premier mass spectrometer (Waters Corporation, Manchester, UK). Peptides were trapped and desalted prior to reverse phase separation using a Symmetry C18 5 *µ*m, 5 mm x 300 *µ*m pre-column. Peptides were then separated prior to mass spectral analysis using a 10 cm x 75 µm C18 reverse phase analytical column. Mass accuracy was maintained during the run using a lock spray of the peptide glu-fibrinopeptide B delivered through the auxiliary pump of the NanoAcquity at a concentration 200 fmol/ µl and at a flow rate 500 nl/min. The LC-MS^E^ method acquires precursor and product ion data on all charge-states of an eluting peptide across its entire chromatographic peak width, providing more comprehensive precursor and product ion spectra. Peptides were analyzed in positive ion mode using a Q-ToF Premier mass spectrometer that was operated in v-mode with the resolving power of 10 000 fwhm. Prior to analyses, the ToF analyzer was calibrated using the doubly charge of glu-fibrinopeptide B (785.8426 m/z). Post calibration data files were corrected using the doubly charged precursor ion of glu-fibrinopeptide B (785.8426 m/z) with a sample frequency of 30 s. Accurate mass LC-MS data were collected in a data-independent and alternating low and high collision energy mode. The spectral acquisition time in each mode was 1 s with a 0.15 s interscan delay. In low energy MS mode, data were collected at constant collision energy of 10 eV. In MS^E^ mode, collision energy was ramped from 15 to 35 eV during each 1s data collection cycle.
